# Association of Systemic Inflammatory Response Index and Prognostic Nutritional Index Scores with Sarcopenia in Patients with Metastatic Gastric Cancer

**DOI:** 10.3390/medicina61050785

**Published:** 2025-04-23

**Authors:** Busra Kanbur, Ilkay Tugba Unek, Mehmet Uzun, Caner Ozturk, Raif Can Yarol, Ali Balci

**Affiliations:** 1 Department of Internal Medicine, Savastepe State Hospital, Balikesir 10580, Turkey; 2 Department of Medical Oncology, Dokuz Eylul University, Izmir 35340, Turkey; ilkaytugbaunek@gmail.com; 3 Department of Medical Oncology, Kahramanmaras Necip Fazil City Hospital, Kahramanmaras 46050, Turkey; memed.uzun3846@gmail.com; 4 Department of Radiology, Dokuz Eylül University, Izmir 35340, Turkey; cozturk95@gmail.com (C.O.); raifyarol@gmail.com (R.C.Y.); alibalciradyolog@yahoo.com (A.B.)

**Keywords:** gastric cancer, sarcopenia, SMI, inflammatory biomarkers

## Abstract

*Background and Objectives:* Sarcopenia is frequently observed in cancer patients and is associated with short survival. In this study, the aim was to research the sarcopenia risk factors, the correlation of sarcopenia with inflammatory biomarkers, and the prognostic significance of sarcopenia and inflammation markers in patients with metastatic gastric cancer. *Material and Method*: The study included 177 patients diagnosed with metastatic gastric cancer attending Dokuz Eylül University Faculty of Medicine (DEUFM) Medical Oncology clinic from 2016 to 2022. The skeletal muscle area at L3 vertebral level was identified on abdominal computed tomography (CT) images, and the skeletal muscle index (SMI, cm^2^/m^2^) was calculated. Additionally, PLR, MLR, NLR, dNLR, SIRI, SII, PIV, PNI, CAR, and LAR were assessed among systemic inflammatory biomarkers. Cut-off values were determined with ROC curve analysis. Survival analyses were performed with the Kaplan–Meier method, and risk factors were investigated with Cox regression analysis. For all statistical analyses, *p* < 0.05 was accepted as significant. *Results:* Among patients, 71.8% were identified to have sarcopenia. Significant levels of difference were identified for median SIRI, NLR, MLR, PLR, SII, PNI, and dNLR values between patients with and without sarcopenia (*p* < 0.05). The sarcopenia risk was assessed between groups created according to the cut-off values for inflammation markers. Univariate regression analysis found that SIRI, PIV, NLR, MLR, PLR, SII, PNI, and dNLR were statistically significant (*p* < 0.05). Multivariate analysis identified SIRI and PNI as independent risk factors. For all patients, median overall survival was identified to be 12.4 ± 0.8 months (CI 95%, 10.8–13.9). For patients with sarcopenia, overall survival duration was 11.5 ± 0.8 months, while survival duration for patients without sarcopenia was 17.5 ± 4.6 months (*p* = 0.010). Elevation in the inflammatory biomarkers of SIRI, NLR, SII, LAR, and CAR and low PNI values appear to be associated with short survival (*p* < 0.05). *Conclusions:* In this study, sarcopenia was frequently observed in patients with metastatic gastric cancer and sarcopenia was associated with shorter survival. A significant correlation was observed between sarcopenia and inflammatory biomarkers, with SIRI and PNI identified to be independent risk factors for sarcopenia. Our study emphasizes the prognostic importance of sarcopenia and inflammatory markers for the management of patients with metastatic gastric cancer.

## 1. Introduction

Gastric cancer is the cancer type in the fifth place in terms of incidence and mortality according to Global Cancer Observatory (GLOBOCAN) data from 2022 [1]. In spite of chemotherapy, median survival duration is generally less than 1 year with recurrence/metastatic disease [2]. Apart from several well-known factors affecting prognosis in gastric cancer, a history of weight loss at the time of diagnosis was shown to be associated with poor prognosis. Loss of muscle mass plays an important role in weight loss [3,4].

Sarcopenia is a progressive syndrome of loss of skeletal muscle and muscle strength. The European Working Group on Sarcopenia in Older People (EWGSOP) defined sarcopenia as a function of low muscle mass and low muscle function [5]. In clinical practice, muscle function is rarely evaluated, and sarcopenia is frequently defined in the cancer literature as just having less muscle mass. When sarcopenia is diagnosed by MRI or CT, the most common technique for objective and standardized examination, it is referred to as “radiologically defined sarcopenia” [6]. In recent years, the use of computed tomography (CT) for the detection of sarcopenia and the accuracy of this technique have been widely accepted [7]. Abdominal CT is an imaging method frequently used during diagnosis and follow-up of gastric cancer especially, and the muscle area at the third lumbar vertebra (L3) level on these images is divided by the square of the patient’s height to calculate the third lumbar vertebra skeletal muscle index (L3 SMI cm^2^/m^2^), and this is used to detect sarcopenia [8]. Sarcopenia emerging in oncology patients may be due to the side effects of treatment and has significant effects on patient survival [9]. Recent research showed that sarcopenia is an important risk factor in terms of chemotherapy toxicity, especially in gastric cancer patients, and a poor prognostic factor, and survival durations are reduced in sarcopenic patients [10].

Inflammatory prognostic indexes are formulated by combining parameters like leukocytes, lymphocytes, neutrophils, monocytes, platelets, albumin, c-reactive protein (CRP), and lactate dehydrogenase (LDH). Grivennikov et al. stated that inflammatory responses play determinative roles in different stages of tumor development, including the initiation of tumorigenesis, progression, malignant transformation, and metastasis [11]. Neutrophil to lymphocyte ratio (NLR), platelet to lymphocyte ratio (PLR), and monocyte to lymphocyte ratio (MLR) are commonly used to determine prognosis in gastric cancer, as with many other malignancies [12]. There are studies showing that the systemic inflammation response index (SIRI), calculated with neutrophil, lymphocyte, and monocyte values, is a prognostic marker for gastric cancer [13]. The pan-immune inflammatory value (PIV), calculated with neutrophil, monocyte, platelet, and lymphocyte values, has gained increasing importance in recent times, and studies show that PIV is associated with survival and progression-free survival in cancer patients [14]. The systemic inflammatory index (SII) is calculated with platelet, neutrophil, and lymphocyte values, and patients with low SII were determined to survive for longer durations in some research [15]. The prognostic nutritional index (PNI), used as a simple and applicable nutritional test, is calculated with albumin and lymphocyte values. A study by Ding et al. showed that patients fed inadequately in terms of nutrition had lower tolerance to adverse drug reactions during chemotherapy; this situation affected the continuity of the chemotherapy process and resulted in a weaker response to chemotherapy [16]. Another study proposed that the ratio of CRP to albumin (CAR) was a beneficial biomarker for prognosis in patients with gastric cancer [17]. Nakazawa et al. suggested that variations in the LDH to albumin ratio (LAR) were beneficial to determine prognosis in patients with gastric cancer receiving treatment [18].

Sarcopenia in gastric cancer patients is generally associated with weight loss, incorrect diet, and systemic inflammation. When cancer combines with cachexia, the response to treatment may weaken, and life expectancy may shorten [19]. In the literature, it is widely accepted that inflammatory biomarkers and sarcopenia play a role in determining prognosis and survival in gastric cancer. However, there are only a limited number of studies evaluating sarcopenia and inflammatory biomarkers together in patients diagnosed with gastric cancer. This study aims to investigate the risk factors for sarcopenia in patients with metastatic gastric cancer, the relationship between sarcopenia and inflammatory biomarkers, and the prognostic significance of sarcopenia and inflammation markers. Additionally, it seeks to demonstrate that inflammatory biomarkers may be utilized in the assessment of sarcopenia.

## 2. Materials and Methods

### 2.1. Patient Selection and Data Collection

The study involved 177 patients who presented to the DEUFM Medical Oncology Clinic with a diagnosis of gastric adenocarcinoma between 1 December 2016 and 31 December 2022. Eligible patients were those who were 18 years or older, had metastatic disease at the time of diagnosis or during follow-up, did not have a history of a second cancer, and had their diagnosis, treatment, and follow-up managed at the DEUFM Medical Oncology Clinic. Patients without recorded abdominal CT imaging at our hospital, whose laboratory data were inaccessible, whose medical records were not available in the oncology outpatient clinic, or whose height and weight information were missing, were excluded from the study. It was ensured that the CT scans were performed at the time of diagnosis. The demographic and clinical data of patients were retrospectively recorded.

### 2.2. Assessment of Sarcopenia

**Figure 1:**
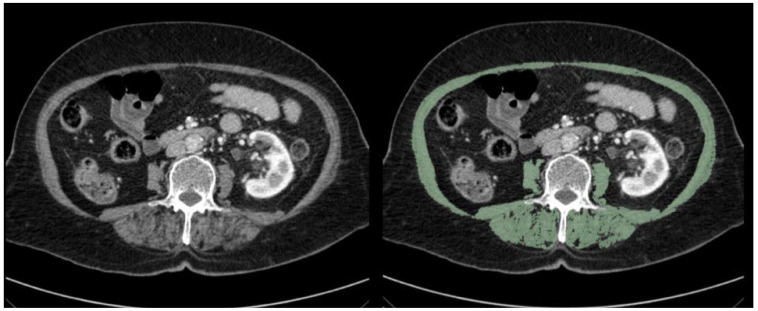
CT L3 Vertebra Level Skeletal Muscle Area Calculation: In the CT image on the left, the L3 vertebra level before scanning is shown using the 3D-Slicer application, while the green-shaded areas in the CT image on the right represent the skeletal muscle areas scanned with 3D-Slicer at the same level.

For the diagnosis and degree of sarcopenia, measurements were performed in the DEUFM Radiology Department. Measurements were conducted using multi-slice CT devices (Aquilion PRIME, Toshiba Medical Systems, Tokyo, Japan; Ingenuity CT, Philips Healthcare, Amsterdam, Netherlands) and 3D-Slicer software (version 5.6.1; www.slicer.org, accessed on 21 April 2025) sourced from Dokuz Eylul University, Izmir, Turkey (Figure 1). At L3 vertebral level, the psoas muscle, paraspinal muscles (erector spinae, quadratus lumborum), and abdominal muscles (transversus abdominis, external and internal oblique muscles, rectus abdominis) were separated from other tissues using Hounsfield unit (HU) values of −29 to +150 and labeled. Muscle contours were manually delineated to prevent measurement errors, and the measurements for all muscle areas included in the section were calculated in cm square (cm^2^). Later, the total muscle area obtained was divided by the square of the patient’s height to calculate the skeletal muscle index (SMI). In our study, due to the geographical location of our country, the L3 SMI cut-off values defined by the European Working Group on Sarcopenia in Older People (EWGSOP) were used for the assessment of sarcopenia. According to the literature data, sarcopenia is identified if the L3 SMI values are below 52.4 cm^2^/m^2^ for men and 38.5 cm^2^/m^2^ for women [8].

### 2.3. Assessment of Inflammatory Biomarkers

Patients had inflammatory biomarkers calculated by using the laboratory results at the time of diagnosis of metastatic disease. The platelet to lymphocyte ratio (PLR), monocyte to lymphocyte ratio (MLR), and neutrophil to lymphocyte ratio (NLR) values were calculated. The derived neutrophil to lymphocyte ratio (dNLR) was calculated by dividing the neutrophil value by the difference between leukocytes and neutrophils. The systemic inflammation response index (SIRI) was calculated by multiplying the neutrophil and monocyte values and dividing by the lymphocyte value. The systemic immune inflammation index (SII) was obtained by multiplying the ratio of neutrophils to lymphocytes by the platelet value. The pan-immune inflammation value (PIV) was calculated by multiplying the neutrophil, monocyte, and platelet values and dividing the result by the lymphocyte value. The prognostic nutritional index (PNI) was obtained by adding 10 times the albumin value (g/dL) to 0.005 times the lymphocyte count (10^3^/ μL). The c-reactive protein (CRP) value was divided by the albumin value to obtain CAR, and the LDH enzyme to albumin ratio was used to obtain the LAR value.

### 2.4. Statistical Analysis

Statistical analyses were performed using the SPSS 22.0 program. After calculating descriptive statistics, the normal distribution of continuous variables was checked with the Kolmogorov–Smirnov and Shapiro–Wilk tests. Comparison of continuous variables in the sarcopenia groups used the Mann–Whitney U test and Student T test, while a comparison of categorical variables used the chi-square and Fisher’s exact test. Results are presented as median (min–max), mean ± sd, and *n* (%). The predictive values of systemic inflammatory biomarkers for sarcopenia were determined with ROC analysis. For area under the curve values (AUC), parameters with *p* value < 0.05 had cut-off values determined using the Youden index (YI = (specificity + sensitivity) − 1). The sensitivity and specificity values were calculated for these cut-off values. Patients were grouped as low and high risk according to these cut-off values. To determine sarcopenia risk factors in these groups, univariate and multivariate regression analyses were performed. The Kaplan–Meier analysis was used for survival analysis related to sarcopenia and systemic inflammatory biomarkers. Overall survival durations for the risk groups for each parameter were compared using the log-rank test. Results are presented with a 95% confidence interval. The statistical significance was accepted as *p* < 0.05.

## 3. Results

### 3.1. Patient Characteristics

The study included 53 women and 124 men for a total of 177 patients; their demographic and clinical characteristics are shown in Table 1. The median body mass index (BMI) of patients was 22.9 (12.5–53.2), and the median SMI score was 42.1 (19.1–42.6). For men, median SMI was 44.2 (19.1–61.8), while for women, median SMI was 37.5 (22.2–75.0). Among patients, 71.8% were diagnosed with sarcopenia.

### 3.2. Demographic and Clinical Characteristics of Patients According to the Presence of Sarcopenia

When the demographic and clinical characteristics of patients are compared according to the presence of sarcopenia, in the sarcopenia group, 77.2% were men, with the non-sarcopenia group comprising 52.0% men (*p* = 0.001). Comparing those with sarcopenia to those without sarcopenia, median BMI was lower (*p* < 0.001), with a higher proportion of underweight patients and a lower proportion of overweight patients (*p* = 0.023). The proportion of patients with lung metastasis was higher in the sarcopenia group (*p* = 0.045). When the systemic inflammatory biomarkers were evaluated in the two groups, there were statistically significant differences observed for the median SIRI, NLR, MLR, PLR, SII, PNI, and dNLR values (Table 2).

### 3.3. Sarcopenia Risk Factors

**Figure 2:**
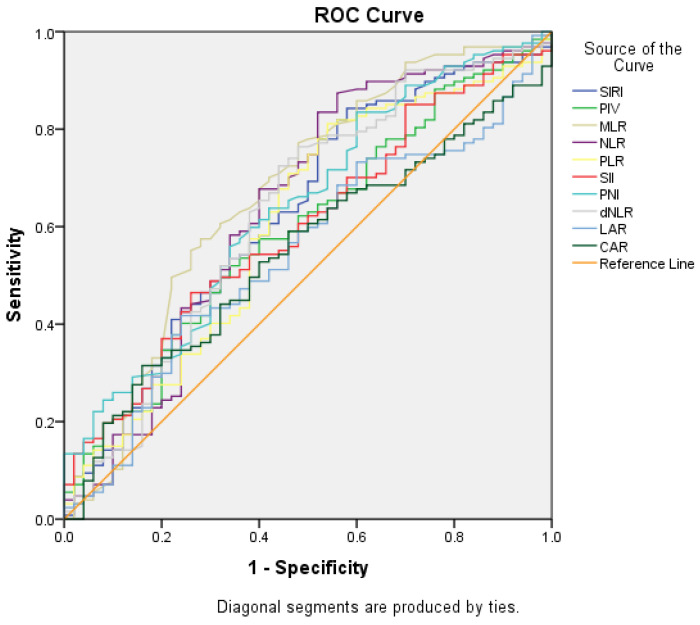
Predictive values of inflammatory biomarkers for sarcopenia (ROC curve).

For the inflammatory biomarkers, with 1.14 cut-off for SIRI, there was 84.3% sensitivity and 42% specificity (AUC = 0.629; *p* = 0.008); with 630.41 cut-off for PIV, there was 52% sensitivity and 66% specificity (AUC = 0.594; *p* = 0.05); with 2.18 cut-off for NLR, there was 87.4% sensitivity and 44% specificity (AUC = 0.641; *p* = 0.004); with 0.43 cut-off value for MLR, thee was 57.5% sensitivity and 72% specificity (AUC = 0.670; *p* < 0.001); with 155.65 cut-off value for PLR, there was 78% sensitivity and 48% specificity (AUC = 0.608; *p* = 0.025); with 1227.5 cut-off value for SII, there was 46.5% sensitivity and 74% specificity (AUC = 0.597; *p* = 0.004); with 1.70 cut-off value for dNLR, there was 76.4% sensitivity and 52% specificity (AUC = 0.631; *p* = 0.007); and with 50.35 cut-off value for PNI, there was 83.5% sensitivity and 40% specificity (AUC = 0.639; *p* = 0.004) for prediction of sarcopenia (Figure 2).

Comparisons between the groups created with the identified cut-off values were performed using univariate logistic regression analysis, and the sarcopenia risk was evaluated between the groups (Table 3). According to the multivariate regression analysis results for the model created with SIRI, SII, PNI, and dNLR, SIRI and PNI were found to be statistically significant risk factors (Table 3).

### 3.4. Factors Determining General Survival

**Figure 3:**
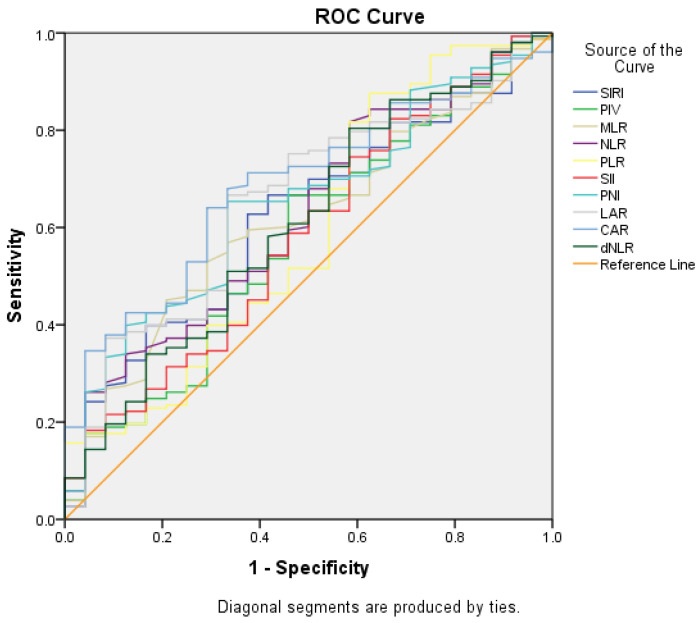
Predictive values of inflammatory biomarkers for overall survival (ROC curves).

For all patients, median overall survival was identified as 12.4 ± 0.8 months (CI 95%, 10.8–13.9). For the inflammatory biomarkers, with 1.77 cut-off value for NLR, there was 62.7% sensitivity and 62.5% specificity (AUC = 0.621; *p* = 0.057); with 2.18 cut-off value for NLR, there was 81.7% sensitivity and 41.7% specificity (AUC = 0.624; *p* = 0.051); with 621.73 cut-off value for SII, there was 74.5% sensitivity and 41.7% specificity (AUC = 0.583; *p* = 0.046); with 46.95 cut-off value for PNI, there was 65.4% sensitivity and 66.7% specificity (AUC = 0.650; *p* = 0.018); with 45.98 cut-off value for LAR, there was 66.7% sensitivity and 66.7% specificity (AUC = 0.653; *p* = 0.016); and with 1.89 cut-off value for CAR, there was 64.1% sensitivity and 70.8% specificity (AUC = 0.687; *p* = 0.003) for the prediction of overall survival (Figure 3).

The inflammatory biomarkers were identified to have predictive value for overall survival according to the cut-off values determined with ROC curves (Figure 3, Table 4).

The overall survival duration for sarcopenic patients was identified as 11.5 ± 0.8 months, while it was 17.5 ± 4.6 months for patients without sarcopenia (*p* = 0.010). Survival durations were statistically significantly longer for patients with SIRI ≤ 1.77 compared to patients with SIRI > 1.77; for patients with PIV ≤ 630.42 compared to patients with PIV > 630.41; for patients with NLR ≤ 2.18 compared to patients with NLR > 2.18; for patients with SII ≤ 621.73 compared to patients with SII > 621.73; for patients with PNI > 46.95 compared to patients with PNI ≤ 46.95; for patients with LAR ≤ 45.98 compared to patients with LAR > 45.98; and for patients with CAR ≤ 1.89 compared to patients with CAR > 1.89. Table 4 shows the effects of sarcopenia and inflammatory biomarkers on overall survival.

## 4. Discussion

In this study, 71.8% of metastatic gastric cancer patients were identified to have sarcopenia (Table 1). When the systemic inflammatory biomarkers were evaluated for the groups with and without sarcopenia, SIRI, PIV, NLR, MLR, PLR, SII, PNI, and dNLR median values were observed to be different at statistically significant levels between the groups (Table 2).

Comparisons between groups created with cut-off values identified according to ROC curves for inflammatory biomarkers were performed using univariate logistic regression analysis, and the sarcopenia risk between the groups was assessed (Figure 2, Table 3). According to the multivariate regression analysis results for the model created with SIRI, SII, PNI, and dNLR, SIRI and PNI were found to be risk factors at statistically significant levels (Table 3).

Sarcopenia is accepted as an indicator of both systemic inflammation and nutritional status in cancer patients. In the literature, several studies have confirmed the role of sarcopenia and inflammatory markers for the prediction of prognosis [20,21].

Patients receiving a diagnosis of gastric cancer have a history of severe weight loss and reductions in skeletal muscle mass, with increasing tumorigenesis and the development of sarcopenia. Studies stated that weight loss shortens the duration of response to treatment and is a poor prognostic factor for survival [22]. In our study, the presence of sarcopenia was researched by calculating SMI from CT images of patients with a metastatic gastric cancer diagnosis, and the overall survival was evaluated according to sarcopenia status. The overall survival of sarcopenic patients was observed to be shorter at a statistically significant level compared to patients without sarcopenia (Table 4).

According to our study data, the median SMI value for men was 44.2 cm^2^/m^2^, while it was 37.5 cm^2^/m^2^ for women, and these were below the normal values stated in the literature for both sexes. In a study of advanced-stage gastric cancer, Lee et al. found that the median SMI values for both men and women were lower than the normal values [23]. This study detected that the median age of sarcopenic patients was 63, and sarcopenia was observed to be more common in elderly patients with gastric cancer. Sarcopenia is thought to be caused by increased tumourigenesis as well as physiological aging. A review of the literature reveals that cancer, and its treatment can raise the incidence of sarcopenia in older persons and exacerbate current muscular loss [24]. In our research, 77.2% of men and 22.8% of women had sarcopenia identified at the time of diagnosis. Among patients with sarcopenia, the rate of male patients was found to be higher by a statistically significant level compared to patients without sarcopenia (77.2% vs. 52.0%; *p* = 0.001). Cancer-related hypogonadism in men is believed to have a worse prognostic impact compared to women, and in patients with advanced-stage cancer, it is associated with low muscle mass and reduced survival. The effectiveness of testosterone replacement in preventing muscle loss remains a topic of debate [25]. Similarly, the median BMI values in the sarcopenia groups and the BMI group distributions were found to be statistically significantly different (*p* < 0.001; *p* = 0.023, respectively). We detected that the median BMI value was identified as 22.9, and the BMI values of patients without sarcopenia were found to be statistically significantly higher compared to those with sarcopenia. In a study of the Chinese population, Yu et al. stated that high BMI values had a preventive effect on sarcopenia according to their findings [26].

When the systemic inflammatory biomarkers are evaluated between the group with sarcopenia and without sarcopenia, median SIRI values were identified to be statistically significantly different between the groups (Table 2, *p* = 0.008). At the same time in our study, multivariate regression analysis found that SIRI was an independent risk factor for sarcopenia (Table 3). A study evaluating the correlation between sarcopenia and SIRI in hepatocellular carcinoma identified that the median survival of the group with low SIRI was 25.0 months, while it was identified as 18.6 months in the group with high SIRI (*p* = 0.001). This study also showed that SIRI and sarcopenia determined the response of patients to the applied treatment [27]. In the literature, there is no study found showing the correlation between sarcopenia and SIRI in metastatic gastric cancer. Our study is the first to show that SIRI value is an independent risk factor for sarcopenia. Our results lead to consideration that the SIRI value, easily measured using routine blood tests, may be a sarcopenia marker in clinical practice. The SIRI value may guide clinicians about earlier recognition of sarcopenia and optimal treatment planning for sarcopenia. The results of our study show that in addition to ensuring early diagnosis of sarcopenia, the SIRI value may be used as a prognostic factor in clinical practice.

In our study, the median values for NLR, dNLR and PLR were identified to be different in a statistically significant way between patients with and without sarcopenia (Table 2). A study of patients with operable gastric cancer identified high NLR and PLR values were associated with sarcopenia and concluded that the systemic inflammatory response increased with the incidence of sarcopenia. Additionally, suspicion of sarcopenia with inflammatory markers like PLR and NLR may assist clinicians about performing nutritional interventions and taking other precautions [28]. When the literature is investigated, no study was encountered showing the correlation of dNLR with sarcopenia. Our study has the feature of being the first study showing dNLR value is associated with sarcopenia.

Univariate analysis in our study showed that patients with MLR > 0.43 had 3.5 times the risk of sarcopenia compared to patients with MLR < 0.43 (Table 3). Similar to our study, Lin et al. showed lymphocyte monocyte ratio (LMR) was an independent risk factor for sarcopenia in gastric cancer and identified that low LMR was associated with poor survival [29].

When the systemic inflammatory biomarkers were evaluated in our research between the group with sarcopenia and the group without sarcopenia, the median values of SII and PNI were identified to be statistically significantly different between the groups (Table 2). At the same time, in our study, multivariate regression analysis showed that PNI was an independent risk factor for sarcopenia (Table 3).

A study including patients with local advanced gastric cancer stated that PNI and SII accurately identified sarcopenia and may be used as suitable markers for sarcopenia screening [30].

A study including operated gastric cancer patients identified a correlation between sarcopenia with shorter survival. They emphasized that the identification of body composition changes with CT data, easily obtained in routine clinical practice, and ensuring adherence to treatment with timely nutritional support were important [31]. Similarly, another study evaluated the impact of sarcopenia prior to treatment on survival in patients with gastrointestinal system cancer receiving immunotherapy and found that patients with sarcopenia had significantly shorter survival compared to those without (*p* < 0.001) [32]. We detected that the overall survival duration for patients with sarcopenia was 11.5 ± 0.8 months, while the survival duration for patients without sarcopenia was 17.5 ± 4.6 months (*p* = 0.010). The results of our study show that sarcopenia is associated with shorter survival, supporting the literature on this topic.

In addition to identifying sarcopenia as a poor prognostic factor in our study, elevated SIRI, NLR, SII, LAR, and CAR levels and low PNI value among inflammatory biomarkers were associated with shorter survival. The results of our study are consistent with the results of studies showing a correlation between inflammatory biomarkers and survival in patients with metastatic gastric cancer in the literature [33,34].

In sarcopenic patients, resistance exercise and optimizing nutritional intake are well-established effective interventions. A 2018 international guideline strongly recommends resistance-based training and conditionally advises increasing protein and calorie intake, along with protein supplementation when necessary [35]. A recent multicenter randomized controlled trial demonstrated that a multicomponent intervention, including moderate-intensity exercise and personalized nutritional counseling, reduced the incidence of mobility disability (defined as the inability to walk 400 m in less than 15 min) by 22% in patients with frailty and sarcopenia [36]. Patients presenting at the hospital with any neoplastic pathology should be managed by a member of the oncology team as their expertise allows them to address cachexia, which may result from both the disease and therapies that often reduce appetite and alter taste, leading to an inappropriate caloric intake for the patient [37]. Based on the analysis of the data from our study, we propose that nutrition and exercise planning can positively impact survival and prognosis in patients within the risk groups.

The result of this study investigating sarcopenia in different cancer types emphasizes that due to the prognostic impact of malnutrition and low muscle mass on treatment tolerance, quality of life, and survival, routine screening and assessment of malnutrition should be performed in all cancer patients, particularly in those with tumors located in the gastrointestinal, pancreatic, head and neck, and lung regions [38]. Additionally, a review of the literature has revealed that in head and neck cancers, the incidence of any degree of radiotherapy-related toxicity is significantly associated with low SMI [39].

The retrospective and single-center nature of data collection, the limited number of patients, and the inability to assess muscle function in sarcopenia grading due to most patients being deceased posed limitations to our study. However, the strength of the study lies in the sarcopenia grading being conducted collaboratively by three radiologists, along with the incorporation of numerous inflammatory biomarkers that were not previously investigated to support the diagnosis of sarcopenia.

## 5. Conclusions

In conclusion, in this study, sarcopenia was observed frequently in patients with metastatic gastric cancer, and sarcopenia was associated with shorter survival. Significant correlations were observed between sarcopenia and inflammatory biomarkers, while SIRI and PNI were identified to be independent risk factors for sarcopenia. Our results lead to the consideration that SIRI and PNI values, easily measured with routine blood tests, may be sarcopenia markers in clinical practice and guide clinicians in earlier recognition of sarcopenia and optimal treatment planning for sarcopenia. Our study emphasizes the prognostic importance of sarcopenia and inflammatory markers during the management of patients with metastatic gastric cancer.

## Figures and Tables

**Table 1:** Baseline characteristics of patients.

Parameters		*n* (%)
Median age, years (min–max)		58 (26–84)
Age group	<60 years	72 (40.7)
	>60 years	105 (59.3)
Sex	Woman	53 (29.9)
	Man	124 (70.1)
ECOG	0–1	148 (83.6)
	2–3	29 (16.4)
Histopathological type	Adenocarcinoma	118 (66.7)
	Signet ring	58 (32.8)
	Adenosquamous	1 (0.6)
BMI groups	Underweight (<18.5 kg/m^2^)	16 (9)
	Normal (≥18.5 and <25 kg/m^2^)	106 (59.9)
	Overweight (≥25 and <30 kg/m^2^)	43 (24.3)
	Obese (≥30 kg/m^2^)	12 (6.8)
Sarcopenia	Yes	127 (71.8)
	No	50 (28.2)
Metastasis at diagnosis	Yes	125 (70.6)
	No	52 (29.4)
Liver metastasis		75 (42.4)
Lung metastasis		43 (24.3)
Bone metastasis		38 (21.5)
Peritoneal metastasis		126 (71.2)
Distant lymph node metastasis		82 (46.3)
Other metastasis		70 (39.5)

BMI: body mass index.

**Table 2:** Baseline characteristics of patients stratified by sarcopenia.

		Sarcopenia (*n* = 127)	No Sarcopenia (*n* = 50)	*p*
Median age, years (min–max)		63 (31–84)	59 (26–84)	0.074
Age group	≥60.0 years	46 (36.2)	26 (52)	0.054
	<60.0 years	81 (63.8)	24 (48)	
Sex	Man	98 (77.2)	26 (52.0)	**0.001**
	Woman	29 (22.8)	24 (48.0)	
ECOG	2–3	106 (83.5) 106 (83.5)	42 (84) 42 (84)	0.931
	0–1	21 (16.5)	8 (16)	
BMI		22.3 (12.5–53.2)	24.4 (13.7–35.1)	**<0.001**
BMI group	Normal	80 (63)	26 (52)	**0.023**
	Underweight	15 (11.8)	1 (2)	
	Overweight	25 (19.7)	18 (36)	
	Obese	7 (5.5)	5 (10)	
Metastasis at diagnosis	Yes	90 (70.9)	35 (70)	0.909
	No	37 (29.1)	15 (30)	
Liver metastasis		57 (44.9)	18 (36)	0.282
Lung metastasis		36 (28.3)	7 (14)	**0.045**
Bone metastasis		25 (19.7)	13 (26)	0.347
Peritoneal metastasis		91 (71.7)	35 (70)	0.827
Distant lymph node metastasis		59 (46.5)	23 (46)	0.956
Other metastasis		56 (44.1)	14 (28)	0.06
SIRI		2.4 (0.3–21.9)	1.7 (0.5–20.3)	**0.008**
PIV		637 (30.1–8580.9)	495.6 (62.4–3587.7)	0.051
NLR		3.6 (0.8–39.7)	2.7 (1.0–12)	**0.004**
MLR		0.5 (0.1–1.9)	0.3 (0.1–2)	**<0.001**
PLR		206.0 (34.1–912.9)	160.7 (65.8–675)	**0.025**
SII		1150 (100.4–8580.9)	874.9 (208–3800)	**0.046**
PNI		44.4 (21.7–61.7)	47.1 (34.9–65.2)	**0.004**
dNLR		2.4 (0.6–23.8)	1.7 (0.8–7.8)	**0.007**
LAR		52.8 (27.8–2074.3)	49.2 (29.1–468.2)	0.343
CAR		2.9 (0.1–80.6)	2.2 (0.2–107.5)	0.327

BMI: body mass index; SIRI: systemic inflammatory response index; PIV: pan-immune inflammatory value; NLR: neutrophil to lymphocyte ratio; MLR: monocyte to lymphocyte ratio; PLR: platelet to lymphocyte ratio; SII: systemic inflammatory index; PNI: prognostic nutritional index; dNLR: derivative neutrophil to lymphocyte rate; LAR: LDH to albumin ratio; CAR: CRP to albumin ratio; *p*-values indicating statistical significance are denoted in bold.

**Table 3:** Univariate and multivariate regression analyses for the risk of sarcopenia.

	Univariate Analysis			Multivariate Analysis		
	OR	CI 95%	*p*	OR	CI 95%	*p*
**Age**						
<60 years vs. ≥60 years	1.9	1.0–3.7	0.056			
**Sex**						
Woman vs. man	3.1 1.63.1	1.6–6.2 1.6–6.2	**0.001** **0.001**			
**BMI**			**0.040**			
Obese vs. normal	2.2	0.6–7.5	0.210			
Obese vs. underweight	10.7	1.0–109.8	**0.046**			
Obese vs. overweight	1.0	0.3–3.6	0.990			
**Lung metastasis**						
None vs. present	2.4	1.0–5.9	**0.050**			
**SIRI**						
≤1.14 vs. >1.14	3.9	1.9–8.1	**<0.001**	**2.4**	1.0–5.9	**0.044**
**PIV**						
≤630.41 vs. >630.41	2.1	1.1–4.1	**0.033**			
**NLR**						
≤2.18 vs. >2.18	5.5	2.5–11.7	**<0.001**			
**MLR**						
≤0.43 vs. >0.43	3.5	1.7–7.1	**0.001**			
**PLR**						
≤155.65 vs. >155.65	3.1	1.6–6	**0.001**			
**SII**						
≤1227.50 vs. >1227.50	2.6	1.3–5.3	**0.010**	1.0	0.4–2.5	0.979
**PNI**						
≥50.35 vs. <50.35	3.4	1.6–7.0	**0.001**	**2.4**	1.1–5.4	**0.034**
**dNLR**						
≤1.70 vs. >1.70	3.5	1.8–7.0	**<0.001**	1.8	0.7–4.6	0.194

BMI: body mass index; SIRI: systemic inflammatory response index; PIV: pan-immune inflammatory value; NLR: neutrophil to lymphocyte ratio; MLR: monocyte to lymphocyte ratio; PLR: platelet to lymphocyte ratio; SII: systemic inflammatory index; PNI: prognostic nutritional index; dNLR: derived neutrophil to lymphocyte ratio; CI: confidence interval; OR: odds ratio; *p*-values indicating statistical significance are denoted in bold.

**Table 4:** Effects of sarcopenia and inflammatory biomarkers on overall survival (Kaplan–Meier analysis).

	mOS	95% CI	*p* Value
**Sarcopenia**			**0.010**
Yes	11.5 ± 0.8	9.8–13.3	
No	17.5 ± 4.6	8.5–26.4	
**SIRI**			**<0.001**
≤1.77	18.5 ± 2.1	14.3–22.6	
>1.77	9.7 ± 1.2	7.3–12.1	
**NLR**			**<0.001**
≤2.18	23.0 ± 2.3	18.5–27.5	
>2.18	10.8 ± 1.2	8.5–13.0	
**SII**			**0.016**
≤621.73	18.0 ± 4.1	10.0–26.1	
>621.73	11.5 ± 1.0	9.6–13.4	
**PNI**			**<0.001**
≥46.95	16.2 ± 2.5	11.3–21.1	
<46.95	10.4 ± 1.3	7.9–13.0	
**LAR**			**<0.001**
≤45.98	19.5 ± 1.2	17.3–21.8	
>45.98	9.4 ± 1.1	7.2–11.6	
**CAR**			**<0.001**
≤1.89	22.4 ± 2.2	18.1–26.6	
>1.89	9.7 ± 1.2	7.5–12.0	

SIRI: systemic inflammatory response index; NLR: neutrophil to lymphocyte ratio; SII: systemic inflammatory index; PNI: prognostic nutritional index; LAR: LDH to albumin ratio; CAR: CRP to albumin ratio; mOS: median overall survival; CI: confidence interval; *p*-values indicating statistical significance are denoted in bold.

## Data Availability

All data generated or analyzed during this study are included in this published article.

## Abbreviations

The following abbreviations are used in this manuscript:

BMI	Body Mass Index
CAR	CRP Albumin Ratio
CT	Computed Tomography
DEUFM	Dokuz Eylül University Faculty of Medicine
EWGSOP	European Working Group on Sarcopenia in Older People
GLOBOCAN	Global Cancer Observatory
dNLR	Derivative Neutrophil to Lymphocyte Rate
LAR	LDH to Albumin Ratio
MLR	Monocyte to Lymphocyte Ratio
NLR	Neutrophil to Lymphocyte Ratio
PIV	Pan-Immune Inflammatory Value
PLR	Platelet to Lymphocyte Ratio
PNI	Prognostic Nutritional Index
SII	Systemic Inflammatory Index
SIRI	Systemic Inflammatory Response Index
SMI	Skeletal Muscle Index
